# Ultrasound-Assisted Distal Radius Fracture Reduction

**DOI:** 10.7759/cureus.674

**Published:** 2016-07-07

**Authors:** Steve Socransky, Andrew Skinner, Mark Bromley, Andrew Smith, Alexandre Anawati, Jeff Middaugh, Peter Ross, Paul Atkinson

**Affiliations:** 1 Emergency Medicine, Northern Ontario School of Medicine; 2 Emergency Medicine, University of British Columbia Vancouver; 3 Emergency Medicine, University of Calgary; 4 Emergency Medicine, Memorial University of Newfoundland; 5 Emergency Medicine, Saint John Regional Hospital / Dalhousie University; 6 Emergency Medicine, Saint John Regional Hospital; 7 Emergency Medicine, Dalhousie University

**Keywords:** pocus, distal radius, fracture, reduction

## Abstract

Introduction

Closed reduction of distal radius fractures (CRDRF) is a commonly performed emergency department (ED) procedure. The use of point-of-care ultrasound (PoCUS) to diagnose fractures and guide reduction has previously been described. The primary objective of this study was to determine if the addition of PoCUS to CRDRF changed the perception of successful initial reduction. This was measured by the rate of further reduction attempts based on PoCUS following the initial clinical determination of achievement of best possible reduction.

Methods

We performed a multicenter prospective cohort study, using a convenience sample of adult ED patients presenting with a distal radius fracture to five Canadian EDs. All study physicians underwent standardized PoCUS training for fractures. Standard clinically-guided best possible fracture reduction was initially performed. PoCUS was then used to assess the reduction adequacy. Repeat reduction was performed if deemed indicated. A post-reduction radiograph was then performed. Clinician impression of reduction adequacy was scored on a 5 point Likert scale following the initial clinically-guided reduction and following each PoCUS scan and the post-reduction radiograph.

Results

There were 131 patients with 132 distal radius fractures. Twelve cases were excluded prior to analysis. There was no significant difference in the assessment of the initial reduction status by PoCUS as compared to the clinical exam (mean score: 3.8 vs. 3.9; p = 0.370; OR 0.89; 95% CI 0.46 to 1.72; p = 0.87). Significantly fewer cases fell into the uncertain category with PoCUS than with clinical assessment (2 vs 12; p = 0.008). Repeat reduction was performed in 49 patients (41.2%). Repeat reduction led to a significant improvement (p < 0.001) in the PoCUS determined adequacy of reduction (mean score: 4.3 vs 3.1; p < 0.001). In this group, the odds ratio for adequate vs. uncertain or inadequate reduction assessment using PoCUS was 12.5 (95% CI 3.42 to 45.7; p < 0.0001). There was no significant difference in the assessment of reduction by PoCUS vs. radiograph.

Conclusions

PoCUS-guided fracture reduction leads to repeat reduction attempts in approximately 40% of cases and enhances certainty regarding reduction adequacy when the clinical assessment is unclear.

## Introduction

The reduction of fractures is a commonly performed procedure in emergency departments (EDs). In most Canadian EDs, reductions are performed by emergency physicians (EPs). The reduction of distal radius fractures is one of the most commonly performed reductions. Fracture reduction is time-consuming with several steps required: initial evaluation, including X-ray, assembly of equipment and personnel, sedation and/or local anesthesia, reduction attempt(s) and immobilization, and post-reduction X-rays with subsequent patient reassessment. The time taken to complete these steps may have a negative effect on ED patient throughput. Subsequent to the reduction attempt(s), the patient is sent for X-ray often with uncertainty regarding the reduction success. Fluoroscopy is generally not an option for the EP in evaluating the accuracy of reduction. If the reduction is not adequate, further reduction attempts are needed. This utilizes more resources in the ED, orthopedic clinic, or operating room, depending on where further reduction attempts are made.

**Figure 1:**
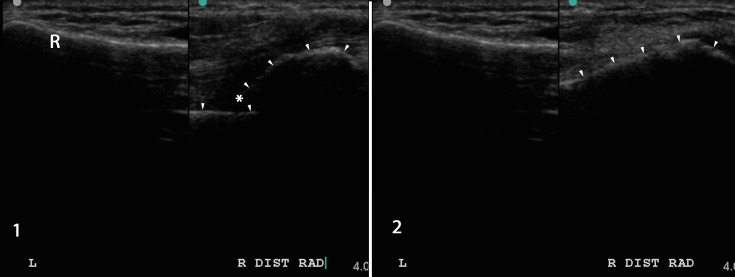
PoCUS-guided fracture reduction. Pre-reduction (1) and post-reduction (2) dual screen views of the normal left and abnormal right distal radius (R).

Point-of-care ultrasound (PoCUS) in Canada has become a well-established part of emergency medical practice [1]. A growing body of literature has demonstrated that PoCUS is useful in the diagnosis and reduction of fractures in adults and children [2-12]. Similar to fluoroscopy, PoCUS may represent a fast and accurate method of determining successful fracture reduction. Unlike fluoroscopy, PoCUS is available immediately in the ED. PoCUS may also obviate the need for the post-reduction X-ray, particularly in the setting where the patient will have yet another X-ray at the time of orthopedic follow-up to evaluate for interval loss of reduction (Figure 1). 

The primary research question for this study was “Does the addition of PoCUS to clinical assessment alter the impression of successful reduction for distal radius fractures in adults?”.

The secondary question was “How does PoCUS compare with clinical assessment and post-reduction X-ray in the assessment of distal radius fracture reduction adequacy?”.

## Materials and methods

### Study design

We performed a multicenter prospective cohort study, using a convenience sample of adult ED patients presenting with a distal radius fracture. The study took place from December 2011 to October 2013. Research Ethics Board approval was obtained from the following institutions: St. Paul’s Hospital (Vancouver, British Columbia), Foothills Medical Centre (Calgary, Alberta), Health Sciences North (Sudbury, Ontario), Saint John Regional Hospital (Horizon Health Network, Saint John, New Brunswick), and the Health Sciences Centre (St. John’s, Newfoundland and Labrador). Approval was obtained from the Research Ethics Boards of all participating hospitals. Horizon Health Network issued approval #HHN 2011-1666.

### Study setting and population

The study took place in the EDs of the following hospitals: St. Paul’s Hospital (Vancouver, British Columbia), Foothills Medical Centre (Calgary, Alberta), Health Sciences North (Sudbury, Ontario), Saint John Regional Hospital (Horizon Health Network, Saint John, New Brunswick), and the Health Sciences Centre (St. John’s, Newfoundland and Labrador). All participating institutions have a high-volume ED and are located in urban settings. All EDs have an emergency medicine residency program recognized by the College of Family Physicians of Canada or the Royal College of Physicians and Surgeons of Canada. Most of the participating study physicians were staff physicians with a minority being emergency medicine residents. All participating physicians performing PoCUS to assess reduction possessed independent practitioner status with the Canadian Emergency Ultrasound Society. All study physicians received didactic and practical training in PoCUS assessment of fractures and guidance of reduction via The EDE 2 Course (www.ede2course.com) or The ECCU Advanced Modules Course (www.emergencyultrasound.ca). For some physicians, this training occurred immediately before the study. In some cases, a study physician, who was the patient’s treating physician, performed both the reduction and PoCUS. In other cases, the treating physician (not necessarily trained in PoCUS for fractures) performed the reduction while a study physician performed the PoCUS.

### Study protocol

Patients with distal radius fractures diagnosed by plain X-ray were considered for study enrollment. Patients were enrolled in the study if all of the following inclusion criteria were met:

1. > 18 years old

2. Able to provide voluntary and informed consent

3. Distal radius fracture was the main and only significant traumatic injury

4. Planned reduction to be performed by the EP

5. Treating EP trained to perform PoCUS for fractures OR one of the study physicians available to perform PoCUS

**Figure 2:**
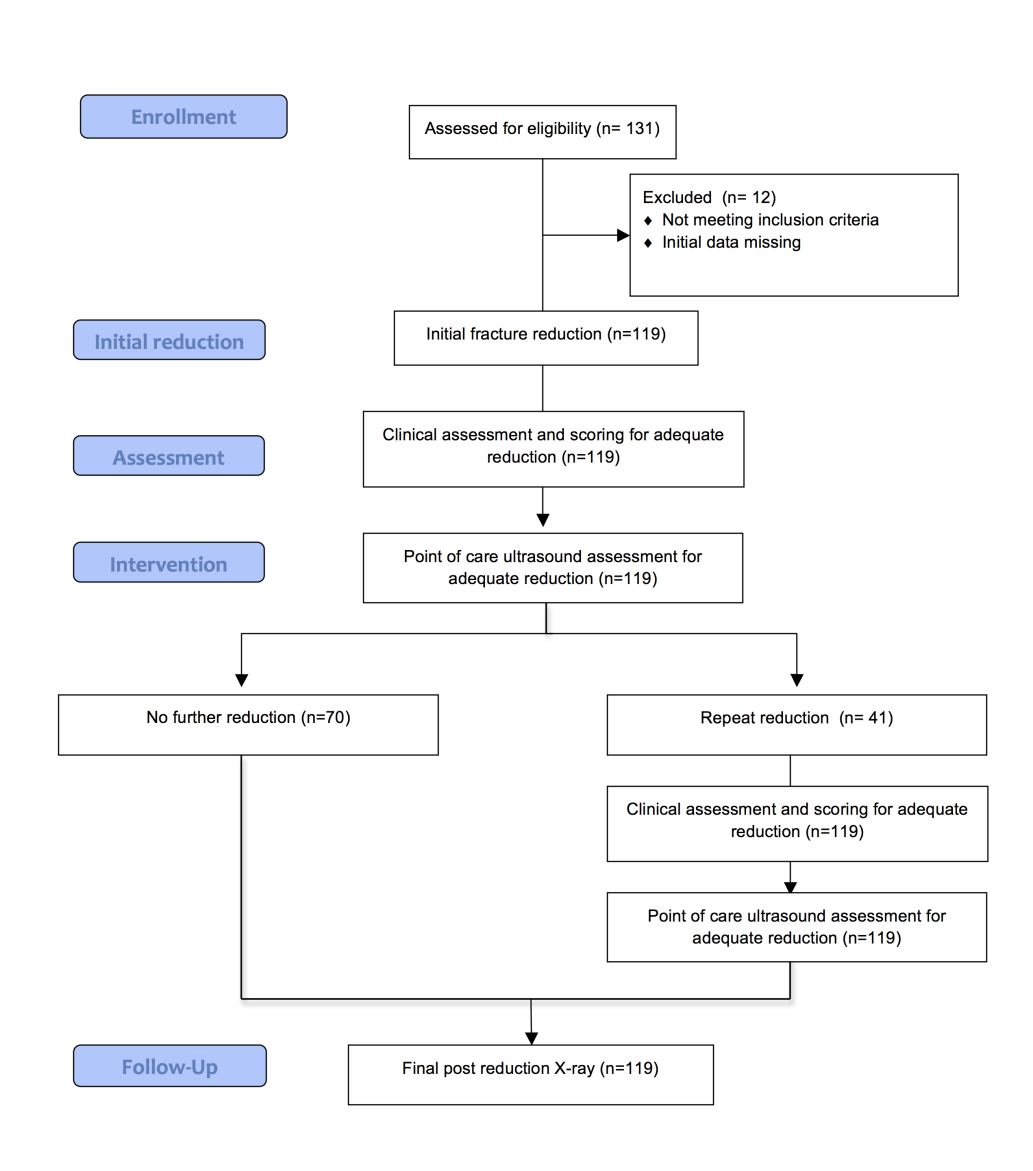
Flow diagram of the study protocol, subjects, and follow-up.

Patients who provided informed and voluntary consent were entered in the following study protocol. Patients were treated as per current practice with the addition of PoCUS as below. No attempt was made to standardize the reduction technique. The key steps are outlined below and are also summarized in Figure 2.

1. Fracture reduction was performed in the usual manner to obtain the best possible reduction, prior to assessment by PoCUS.

2. The treating physician assessed the likelihood of adequate reduction using clinical assessment and recorded this on a standardized form using a Likert scale (1 = definitely NOT adequate, 2 = probably NOT adequate, 3 = uncertain, 4 = probably adequate, and 5 = definitely adequate).

3. Prior to the application of plaster, the study physician evaluated the adequacy of reduction with PoCUS and recorded the results, again on the same Likert scale.

4. Repeat reduction was performed at the discretion of the treating physician. The study physician then reevaluated any repeat reductions with PoCUS and documented their impression yet again. The number of repeat reduction attempts was recorded.

5. The fracture was immobilized when the best reduction possible was obtained.

6. A post-reduction X-ray was obtained and the treating physician recorded the adequacy of reduction on the same Likert scale.

7. The treating physician recorded any repeat reduction attempts that were performed after the post-reduction X-ray.

The following were recorded as measurements or key outcome measures:

1. The difference between the PoCUS and clinical assessment of reduction adequacy following the initial reduction attempt.

2. The rate of repeat reduction attempts based on PoCUS after the best possible reduction was obtained using clinical assessment alone.

3. The improvement in reduction adequacy based on PoCUS vs. clinical assessment following repeat reduction.

### Data analysis

Data was collected using a standardized written form and transferred to a Microsoft Excel spreadsheet. The Wilcoxon signed ranked test was used to compare the overall assessment of reduction by clinical exam vs. PoCUS, as well as the scores on PoCUS and clinical exam before and after repeat reduction. The McNemar’s Chi-square test was used to determine if there was a difference in the number of cases classed in the uncertain category after the initial reduction by clinical exam vs. PoCUS. A Wilcoxon two-sample test was used to compare the difference between PoCUS and clinical assessments. The Kruskal-Wallis test was used to assess for significant differences between centres with respect to the assessment by clinical exam, PoCUS, and X-ray. The Chi-square test was used to assess for differences between centres in the rates of repeat reduction. Descriptive statistics were used elsewhere. It was estimated that for 25% of cases to have a reduction adequacy on PoCUS differing by > 1 point on the Likert scale compared to clinical assessment, 130 cases would provide a confidence interval of +/- 7.5%.

## Results

There were 131 patients with 132 distal radius fractures requiring reduction enrolled in the study. Twelve cases were removed from study analysis due to illegible or incomplete data entry. In the patient with bilateral fractures, only the right distal radius fracture was retained in the study. This left 119 cases for analysis (Figure 2). There were 24 males and 95 females with a mean age of 58.7 years (range: 18-95). The case breakdown by site was Vancouver: 14, Calgary: 38, Sudbury: 26, Saint John: 18, and St. John’s: 23. In 117 cases, the physician performing the reduction and the PoCUS scan could be determined. In these cases, 66 different physicians performed the reductions and 55 physicians performed the PoCUS scans. In 96 cases (82.1%), the same physician performed the reduction and PoCUS. A physician other than an author performed the reduction in 90 cases (77.0%) and did the PoCUS scan in 81 cases (69.2%).

There was no significant difference in the assessment of initial reduction status by PoCUS as compared to the clinical exam (mean score: 3.8 vs. 3.9; p = 0.370; OR 0.89; 95% CI 0.46 to 1.72; p = 0.87) (Table 1, Figure 2). However, when grouped into an outcome of *uncertain or any other category*, significantly fewer cases fell into the uncertain category when assessed by PoCUS than clinically (2 vs.12; p = 0.008).

**Table 1:** Adequacy of Initial Fracture Reduction Based on Clinical Exam and PoCUS

	Clinical assessment	PoCUS assessment
1-Definitely not adequate	2	7
2-Probably not adequate	7	14
3-Uncertain	12	2
4-Probably adequate	86	53
5-Definitely adequate	12	43
Mean Score	3.8	3.9

Repeat reduction was performed on 49 (41.2%) of the 119 patients. In 40 cases, a single repeat reduction attempt was made, with two attempts in eight cases, and three attempts in one case. Repeat reduction was performed in 21 cases where PoCUS assessment indicated that the reduction was probably adequate. Repeat reduction was performed in six cases where PoCUS indicated that the initial reduction was definitely adequate.

Patients who underwent repeat reduction had a lower initial reduction adequacy than patients who did not have a repeat reduction as scored by PoCUS (mean score: 2.6 vs. 4.5; p = 0.009) and as scored clinically (mean: 3.6 vs 4.0; p < 0.001). In patients who did not undergo repeat reduction, the initial PoCUS adequacy score was significantly higher than the clinical score (mean score: 4.5 vs. 4.0; p < 0.001). In patients who did undergo repeat reduction, the initial PoCUS score was significantly lower than the clinical score (mean score: 2.6 vs. 3.6; p < 0.001) (Table 2).

**Table 2:** Likelihood of Repeat Reduction - Clinical Exam vs PoCUS

	No repeat reduction		Repeat reduction	
	Clinical	PoCUS	Clinical	PoCUS
1-Definitely not adequate	1	0	1	7
2-Probably not adequate	2	0	5	14
3-Uncertain	4	1	8	1
4-Probably adequate	54	32	32	21
5-Definitely adequate	9	37	3	6
Mean Score	4	4.5	3.6	2.6

**Figure 3:**
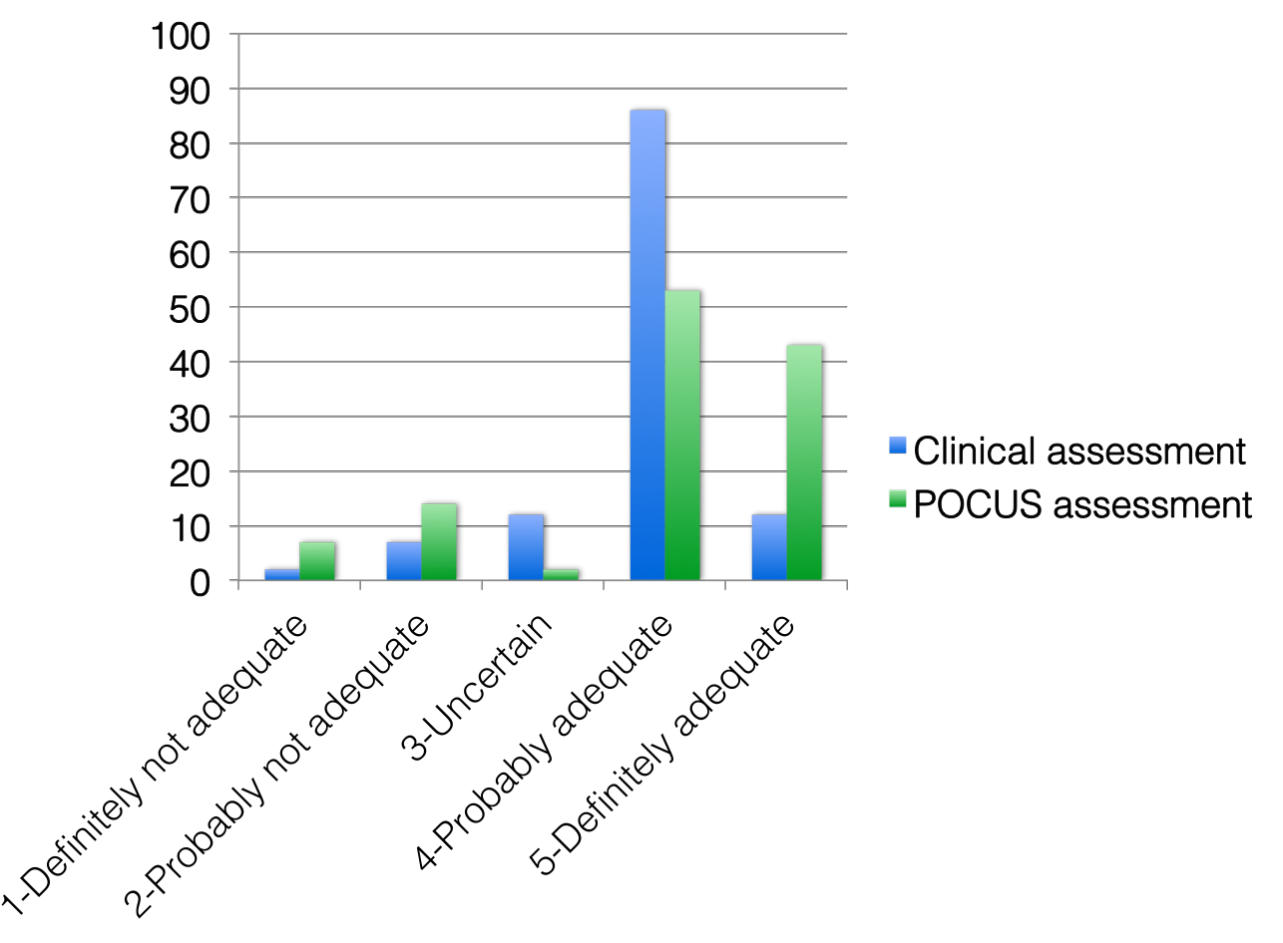
Comparison of initial reduction adequacy scores using point-of-care ultrasound (PoCUS) and clinical assessment.

Repeat reduction led to a significant improvement (p < 0.001) in the PoCUS determined adequacy of reduction (mean score: 4.3 vs 3.1; p < 0.001;) (Table 3, Figure 3). In this group, 46 of 49 patients had an adequate reduction score when assessed by POCUS, compared to 27 of 49 using clinical assessment (OR 12.5; 95% CI 3.42 to 45.7; p < 0.0001).

**Table 3:** Adequacy of Repeat Reduction: Pre- and Post-PoCUS Scores

	Pre-PoCUS	Post-PoCUS
1-Definitely not adequate	7	0
2-Probably not adequate	14	1
3-Uncertain	1	2
4-Probably adequate	21	28
5-Definitely adequate	6	18
Mean Score	3.1	4.3

**Figure 4:**
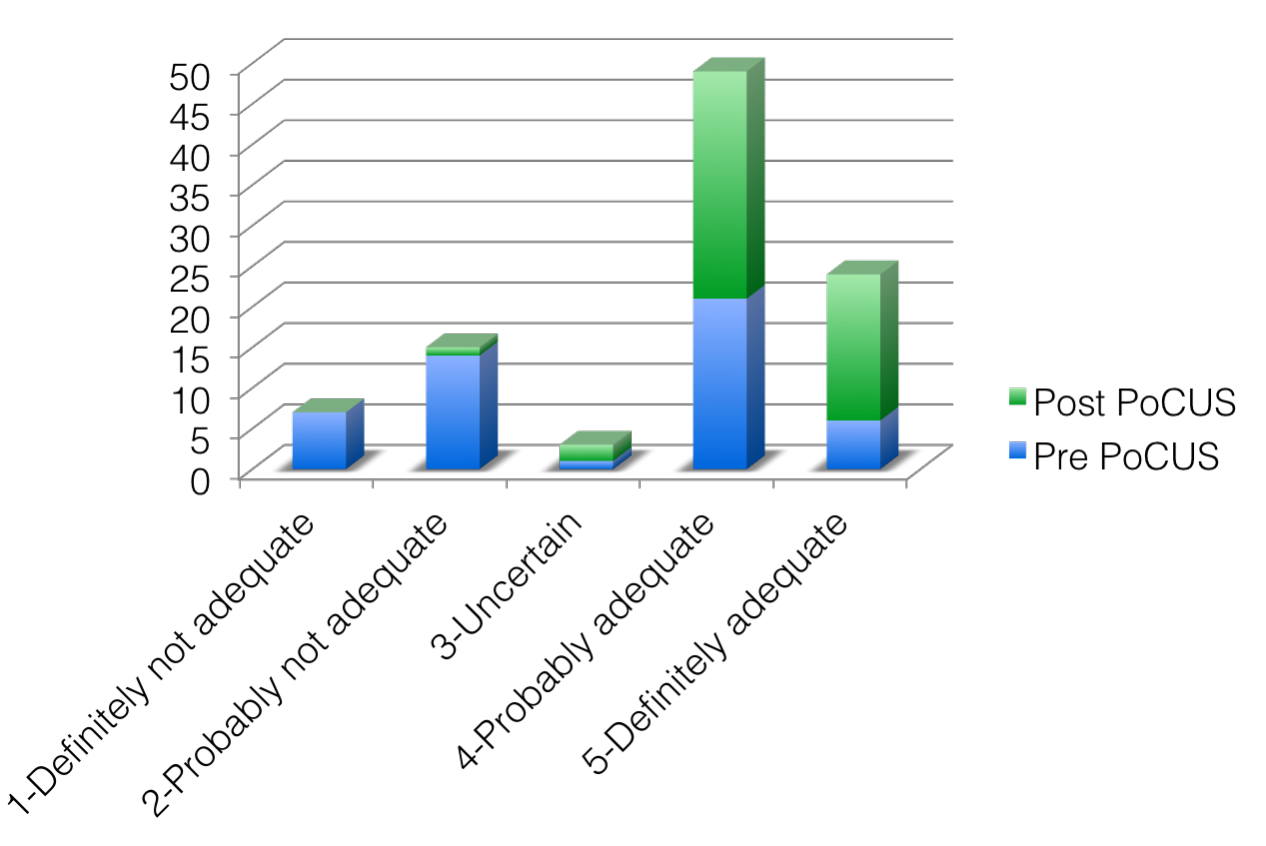
Changes in PoCUS-based adequacy scores for repeat reductions (Pre-reduction PoCUS vs. post-reduction PoCUS).

Data on the adequacy of reduction as determined by the last PoCUS scan compared with the post-reduction X-ray is divided between the groups of patients who did or did not have a repeat reduction attempt. There was no significant difference in the assessment by final PoCUS and final X-ray for patients in either group (No repeat reduction: mean 4.5 vs. 4.6; p = 0.307; repeat reduction: 4.3 vs. 4.3; p = 0.521) (Table 4, Figure 4). 

**Table 4:** Adequacy of Reduction: Last PoCUS vs. X-ray

	No repeat reduction		Repeat reduction	
	Last PoCUS	X-Ray	Last PoCUS	X-Ray
1-Definitely not adequate	0	0	0	2
2-Probably not adequate	0	3	1	4
3-Uncertain	1	1	2	1
4-Probably adequate	32	19	28	11
5-Definitely adequate	37	47	18	31
Mean Score	4.5	4.6	4.3	4.3

## Discussion

In response to our primary question, “Does the addition of PoCUS to clinical assessment alter the impression of successful reduction for distal radius fractures in adults?”, we have shown that the use of PoCUS to guide the reduction of distal radius fractures in the ED leads to additional repeat reduction attempts in approximately 40% of cases following an initial “best possible” reduction as assessed clinically. We have also shown that PoCUS provides greater certainty regarding the initial reduction success than does clinical assessment alone, and that PoCUS and X-ray had similar performance scores for confirmation of post-reduction adequacy. Our findings come from five Canadian EDs where the majority of reductions and PoCUS scans were performed by physicians who were not authors of this study.

Several previous studies have examined the use of ultrasound to diagnosis fractures in adults and children [2-8]. In these studies, ultrasound was performed by various health care practitioners, including emergency physicians, surgeons, radiologists, and cast technicians. The accuracy varied between 86% and 100%, with most in the mid-90% range. The diagnosis was more challenging for smaller bones, undisplaced fractures, and fractures near joints [3, 6].

Ultrasound-assisted fracture reduction has been the subject of several studies [8-12]. Chen, et al. found that pediatric forearm fractures were reduced with an initial success rate of 92% when using ultrasound [8]. Ang, et al. found that ultrasound guidance reduced the need for repeat reduction compared to blind technique as well as a reduced operative rate [9]. Kodama, et al. found similar success rates when comparing ultrasound with fluoroscopy [10]. Chern, et al. and Chinnock, et al. found no difference between ultrasound and X-rays for determining the adequacy of reduction [11-12].

In this study, PoCUS led to a significant increase in certainty regarding the adequacy of reduction versus clinical assessment alone. Once assessed by PoCUS, fewer cases fell into the uncertain category. In addition, PoCUS led to greater certainty regarding the need for repeat reduction. The PoCUS assessment of the adequacy of reduction differed from clinical assessment by 2 or more points on the Likert scale in 14.1% of cases, indicating an inadequate reduction. Repeat reduction was performed in 14 cases (11.7%). In all cases, repeat reduction led to an improvement in adequacy of reduction based on PoCUS. In fact, across all cases where a repeat reduction was performed, the score on PoCUS improved significantly.

Despite the best possible reduction being performed by the treating physician, 49 patients (41.2%) had a repeat reduction performed based on the PoCUS result. Not surprisingly, a lower Likert score on PoCUS was significantly associated with a repeat reduction attempt. In 27 of these 49 cases, the repeat reduction was performed in a patient who was felt to have a reduction that was probably or definitely adequate based on PoCUS. It is unclear why this would be the case. One possibility is that displacement seen on PoCUS (e.g., 1 mm) may not be visible on X-ray. The treating physician may have felt the need to correct this minor displacement despite its unknown clinical significance. Unfortunately, it was not possible to determine the number of patients who would have required repeat reduction attempts following initial post-reduction X-ray if PoCUS had not been used, due to the observational design of the study.

In over 93% of cases, the assessment of the adequacy of reduction by the last PoCUS scan was within 1 point or less on the Likert scale of the assessment by the post-reduction X-ray. However, PoCUS suggested a better adequacy of reduction (by 2 or 3 points on a Likert scale) than X-ray in seven cases (5.8%). In five of these seven cases, the initial clinical assessment of reduction was similar, indicating that the reduction was probably or definitely adequate. While it is possible that the PoCUS scans may have been falsely reassuring in these five cases, in the absence of PoCUS, the treating physician may not have made any further attempts at reduction. In only two of the seven cases was the initial clinical assessment suggestive of an inadequate or uncertain reduction. The initial PoCUS scan yielded the same impression. However, following the repeat reduction attempt, the last PoCUS suggested adequate reduction and may have been falsely reassuring to the treating physician, causing them to make no further attempts at reduction. In all seven cases, had the post-reduction X-ray not been performed, the PoCUS scan may have caused the treating physician to communicate to orthopedics that the fracture was adequately reduced, despite that not being the case.

### Limitations

The observational unblinded nature of this study leaves open the potential for bias in terms of categorizing and scoring the success of each reduction. However, as the best possible reduction was the aim of the intervention, and as there was no parallel control group, it is unlikely that this would have a major influence on the outcomes. Despite attempts at ensuring that training was uniform, some physicians may have had less experience than others in assessing fracture reduction with PoCUS. It is possible that, with continued experience, the number of cases where the PoCUS scan differed from the post-reduction X-ray assessment would be less. It is possible that study physicians assessed adequacy of reduction differently on PoCUS as compared to the post-reduction X-ray. Physicians may have simply looked for reduction of displacement and angulation on PoCUS. However, on X-ray, they may have also used the need for open intraoperative repair to judge whether or not the reduction was adequate or not. Likewise, as specifically commented on by one study physician, the assessment on PoCUS may have been different than the assessment on the post-reduction X-ray due to the loss of reduction between the time of the PoCUS scan and the application of plaster. This is more likely to occur in the case of a fracture that is quite unstable, which are more likely to require open repair.

Although all physicians received similar training, there was no attempt to further standardize the management of fracture reduction, and there may be practice differences between sites in the assessment of reduction adequacy by clinical exam, PoCUS, and post-reduction X-ray. This study did not quantify the degree of displacement or angulation on X-ray or PoCUS. Future research should standardize management and quantify findings on imaging.

## Conclusions

The addition of PoCUS leads to repeat reduction attempts in approximately 40% of distal radius fractures when the best possible reduction has been obtained by clinical assessment alone. PoCUS leads to greater certainty in the assessment of reduction adequacy as compared to clinical assessment only. Although we found that PoCUS and X-ray had similar scores, and the use of PoCUS cannot be recommended to routinely replace the final post-reduction X-ray, it may reduce the requirement for X-ray prior to confirmation of final best reduction; further study is required.
